# Zygoma Bone Shell Technique: A Proof‐of‐Concept Surgical Protocol in Human Cadaver for Bone Reconstruction After Zygomatic Implant Failure

**DOI:** 10.1002/cre2.70093

**Published:** 2025-03-15

**Authors:** Agliardi Enrico Luigi, Pozzi Alessandro, Gherlone Enrico

**Affiliations:** ^1^ Advanced Oral Surgery, Department of Dentistry Vita Salute University, San Raffaele Hospital Milan Italy; ^2^ Department of Clinical Science and Translational Medicine University of Rome Tor Vergata Rome Italy; ^3^ Department of Periodontics and Oral Medicine University of Michigan School of Dentistry Ann Arbor Michigan USA; ^4^ Department of Restorative Sciences Augusta University Augusta Georgia USA; ^5^ Department of Restorative Dentistry and Biomaterials Sciences Harvard School of Dental Medicine Boston Massachusetts USA; ^6^ Department of Dentistry, San Raffaele Hospital Vita Salute University Milan Italy

**Keywords:** implant failure, shell technique, zygoma, zygomatic implants

## Abstract

**Objectives:**

This article aims to present a proof‐of‐concept surgical technique for immediate reconstruction of zygoma anatomy following implant failure and complications, illustrating the related clinical steps in a cadaver specimen. Zygomatic implants represent a paradigm shift, addressing challenges posed by severe maxillary bone atrophy and partial or complete maxillectomy, not suitable for conventional dental implant placement. Despite documented high survival rates, intra‐ and postoperative complications can occur and lead to implant failure, resulting in severe defects extended up to entire height of zygomatic bone pyramid. Such defects may infringe immediate or delayed new implant placement, requiring complex surgical procedures to restore integrity of zygomatic bone anatomy.

**Material and Methods:**

The three‐dimensional reconstruction of zygomatic bone defect was achieved by specific form of guided bone regeneration or shell technique, using a thin cortical plate harvested from external oblique line of the mandible. After a meticulous mechanical debridement of bone defect resulting from implant removal, a thin cortical bone block was harvested from the mandibular ramus. The cleared bone defect was filled with autogenous bone chips and thin bone shell was secured above with a bone fixation screw.

**Results:**

Zygoma Bone Shell technique was able to restore contours of zygomatic pyramid ridge. The comparable composition between mandibular and zygomatic bone, particularly in the cortical region allowed an anatomical resemblance that facilitates optimal structural compatibility, fostering seamless integration of bone graft into zygomatic area.

**Conclusions:**

Within limitations of this proof‐of‐concept, zygoma bone shell technique may offer a viable surgical procedure for immediate bone reconstruction after zygomatic implant failure. Translating the previously reported clinical outcomes of bone shell technique, it may be used same day of failing implant removal to achieve reconstruction of zygomatic anatomy with limited risk of postoperative complications. Further clinical studies are needed to confirm its predictability, reliability and anticipated benefits.

## 1 Introduction

Zygomatic implants (ZI), introduced by Prof. P‐I Brånemark in the late 1980s (Brånemark et al. 2004), represented a paradigm shift in the field of oral and maxillofacial surgery. Initially they were designed to address the intricate challenges posed by severe maxillary bone atrophies and partial or complete maxillectomy defects due to oncologic resection, not suitable for conventional dental implant placement, and providing stable prosthesis retention. The original Brånemark protocol included one implant in each zygoma, traversing the sinus, in combination with two to four anterior conventional implants. In the last two decades, ZI have undergone substantial evolution to accommodate a spectrum of clinical complexities. The quad zygomatic implant concept, where two ZI are inserted on each side, was introduced to provide acceptable antero‐posterior implant positioning for force distribution, in patients without adequate anterior maxillary bone (Lan et al. 2021). Clinical indications were recently reviewed by the ITI consensus (Al‐Nawas et al. 2023) reporting that zygomatic implants are an evidence‐based treatment option to support fixed or removable prostheses and restore partially or completely edentulous maxillae, with high survival rates when splinted to other implants. Zygomatic implants are an alternative when the maxillary bone is completely or partially absent, secondary to benign or malignant tumor resection, trauma or congenital defects, or when the maxillary bone is completely or partially absent, secondary to failure of previously placed implants and/or bone grafts (Al‐Nawas et al. 2023). Nowadays ZI are commonly used to support fixed dental implant prosthesis when traditional dental implants cannot be placed, offering to patients a viable alternative to extensive hard and soft tissue reconstructions (Parel et al. 2001). However, zygomatic implants require advanced surgical skills due to the proximity of vital anatomical structures, such as the orbit, the infratemporal fossa, the infraorbital nerve and the zygomaticofacial nerve and restorative expertise to address all the potential difficulties. Moreover, the placement of long zygomatic implants with limited view of the surgical field and irregular shape of the zygoma can be challenging (Polido et al. 2023).

Although still considered a complex procedure with significant surgical risk and potential for complications, the use of zygomatic implants has grown exponentially, with documented high survival rates, even comparable to conventional implants when used for reconstruction of the atrophic maxilla, as short implants, tilted implants, and implants placed in grafted sinuses (Aparicio et al. 2014; Gracher et al. 2021; Agliardi et al. 2021; Brennand Roper et al. 2023; Agliardi et al. 2023; Del Fabbro et al. 2022). However, intra‐ and postoperative complications can occur, and they demand meticulous attention (Chrcanovic et al. 2016; Moraschini et al. 2023; Vrielinck et al. 2023). The most reported long‐term biological complication was maxillary sinusitis, that may be successfully treated through antibiotics. In the absence of resolution, refractory maxillary sinus infections may need exploration of the patency of the osteo‐meatal complex and other paranasal sinuses. If these therapies are unsuccessful, the ZI may be lost. Oro‐antral fistula, peri‐implant infection of the soft tissues, peri‐implant mucositis and peri‐implantitis, oral vestibular dehiscence, implant fracture, zygomatic bone and orbit fracture, implant overextension, subperiosteal infections due to debris accumulation during site preparation, or local osteonecrosis determined by inadequate flushing of bone debris or excessive implant torque, highlight the critical nature of precise surgical techniques and adherence to established protocols (Kämmerer et al. 2023; Bedrossian and Bedrossian 2018; Tran et al. 2019; Vrielinck et al. 2023). Patient education in oral hygiene maintenance is paramount.

**Figure 1:**
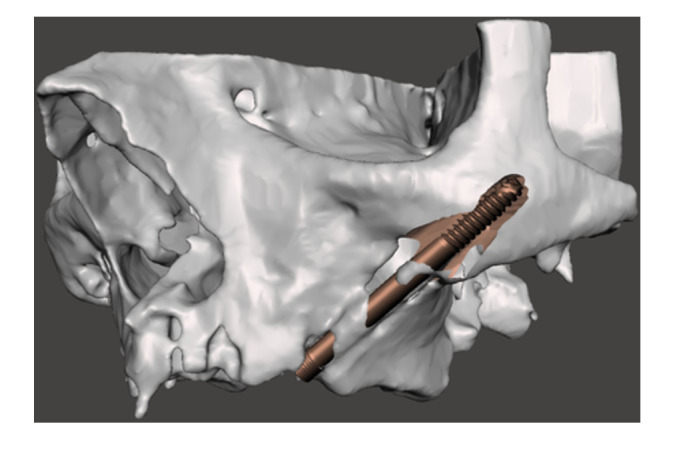
An automatic segmentation algorithm was used to generate an accurate 3D model of the preoperative zygomatic implant failure bone loss.

**Figure 2:**
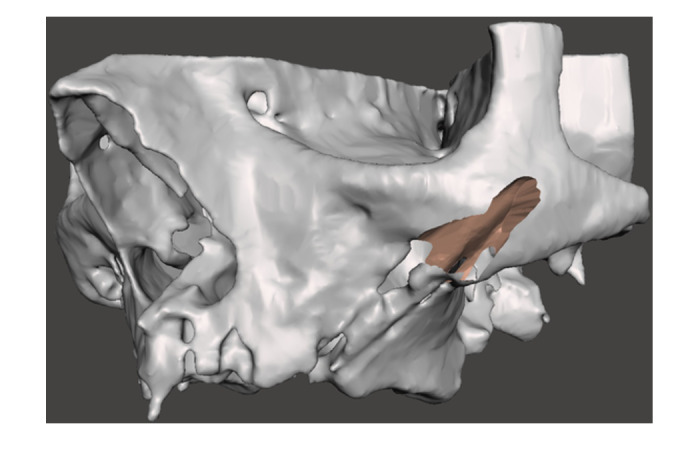
3D rendering simulating bone anatomy deformity residual to the implant removal.

**Figure 3:**
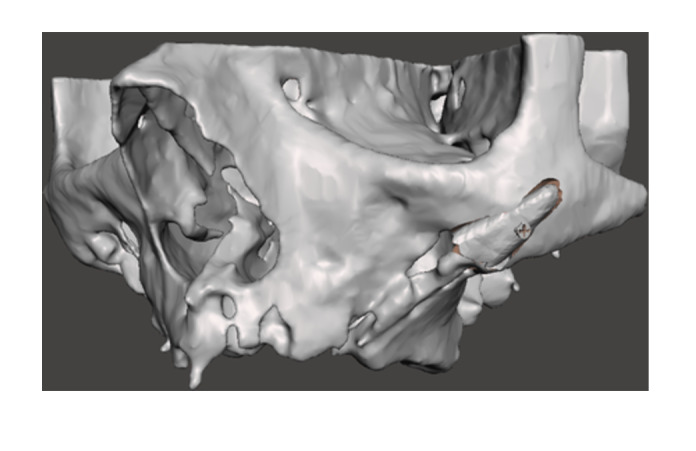
Bone shell rendering to evaluate the shape and dimensions of bone lamina needed to reconstruct the zygomatic anatomy deformity. The lamina was virtually secured with a fixation screw to the residual bone.

Replacing a failing zygomatic implant with a new ZI requires a proper clinical examination, a cone‐beam computer tomography (CBCT) with 3D reconstruction of the area, a detailed assessment of the residual bone anatomy and a rendering of the bone shell dimension, positioning and fixation. (Figures 1, 2, 3) In most of the cases a staged approach is needed because of the presence of bone and soft tissue infection. Indeed, the ZI and even more the quad zygoma failure can result in severe defects extending up to the entire height of the zygomatic bone pyramid which could infringe the immediate or delayed placement of new ZIs, requiring complex surgical procedures to restore the integrity of the zygomatic bone anatomy (Bedrossian and Bedrossian 2018; Tran et al. 2019; Vrielinck et al. 2023; Davó et al. 2024; Xue et al. 2019; Chu et al. 2012; Heredia‐Alcalde et al. 2021; Modabber et al. 2013).

This article aims to present a proof‐of‐concept surgical technique for the immediate reconstruction of zygomatic bone following ZI failure and complications and illustrate the related clinical steps in a cadaver specimen. The protocol should be considered where the failure of zygomatic implants has resulted in a significant destruction of the supporting bone, making the placement of a new implant particularly challenging.

## 2 Methods

The zygomatic bone shell technique is a proof‐of‐concept surgical protocol for immediate bone reconstruction after zygomatic implant failure. The “three‐dimensional” reconstruction or shell technique is a specific form of GBR using a thin cortical plate harvested from the external oblique line of the mandible (Khoury and Hanser 2015). The bone shell surgical technique was introduced to address minor horizontal and vertical bone defects and its favorable application to zygomatic implant is tightly dependent on the extension of the defect. Even though composite bone shells reconstruction with multiple bone laminas may be used in larger defects.

After a meticulous mechanical debridement of the zygomatic bone defect resulted from the removal of the ZI, a thin cortical bone block was harvested from the mandibular ramus. The resulting bone defect was filled with autogenous bone chips and the thin bone shell was secured with a bone fixation screw to restore the contours of the zygomatic pyramid ridge.

### 2.1 Step‐by‐Step Surgical Protocol

#### 2.1.1 Recipient Site Preparation

**Figure 4:**
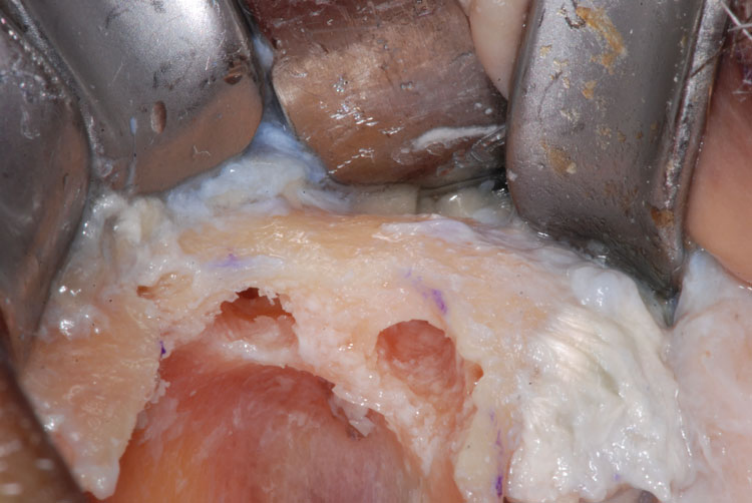
Typical zygoma bone defect resulting from the removal of two zygomatic implants. The residual bone anatomy hampers the immediate and delayed placement of the new zygomatic implant unless zygoma pyramid is not completely restored.

**Figure 5:**
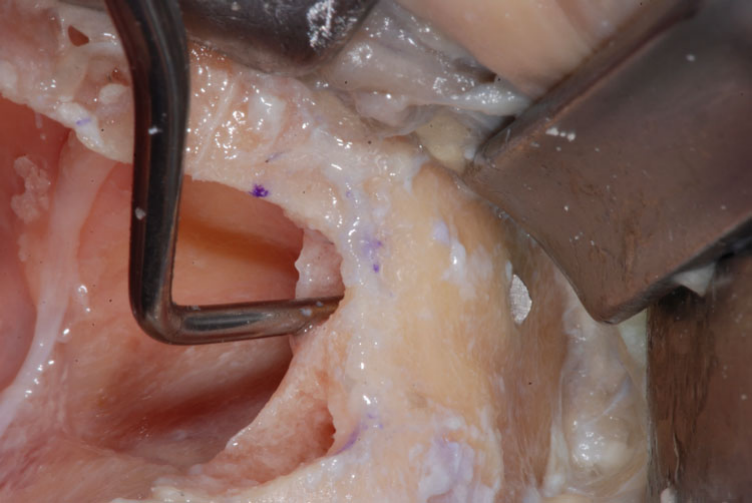
The resulting zygoma bone deformities were mechanically cleared by any debris and remnants with a surgical curette.

**Figure 6:**
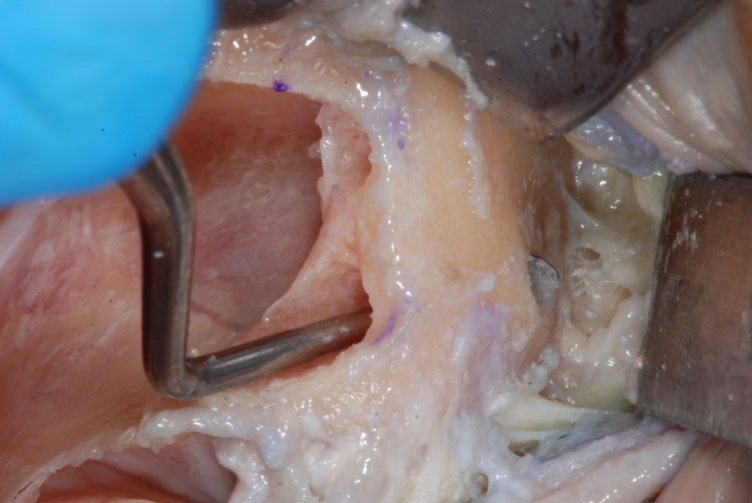
The defect was cleared up to the lateral face of the zygomatic bone, where the periosteum was properly elevated to have a clear view of the tip of the surgical curette. The recipient sites were extensively irrigated with saline and antibiotic solutions.

The failed zygomatic implants were removed, and the resulting bone deformities mechanically cleared by any debris and remnants with a surgical curette (Lucas‐Martin Bone Curette, KLS Martin, Germany). The recipient sites were extensively irrigated with saline solution and antibiotic solution (Rifampicin Ready Made Solution 50 mg/mL) (Figures 4, 5, 6).

#### 2.1.2 Harvesting Procedure

The harvesting area was selected after a careful CBCT assessment of the volume of the zygomatic bone and the area of the buccal shell and mandibular ramus.

An Artificial Intelligence (AI) driven software (Iconix, Xnav Technologies, USA) for automatic segmentation of CBCT data was used to generate an accurate 3D model (Carosi et al. 2022) of the preoperative zygomatic implant failure bone loss (Tao et al. 2023). The 3D rendering was used to simulate bone anatomy deformity residual to the implant removal and, the bone shell shape and dimensions needed to reconstruct the zygomatic anatomy morphology. Software allowed to plan for the bone shell fixation screw placement through the residual zygomatic bone.

**Figure 7:**
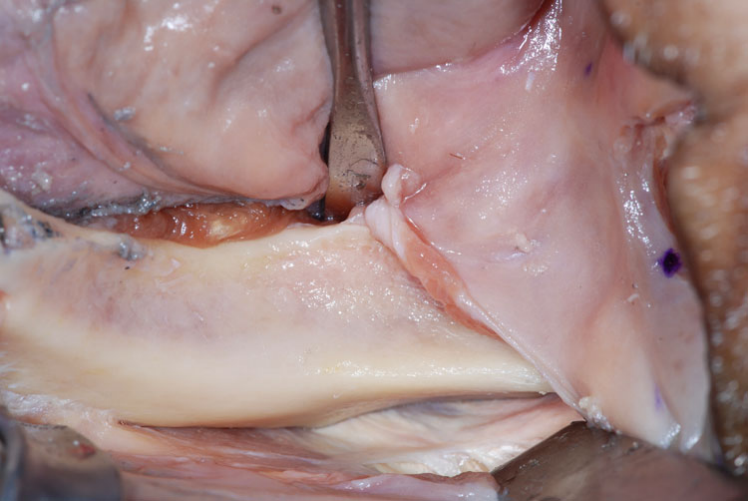
The harvesting area was selected after a careful assessment on the CBCT. The area was widely exposed after the reflection of a mucoperiosteal flap, from the first molar to all extension external oblique line of the mandible.

**Figure 8:**
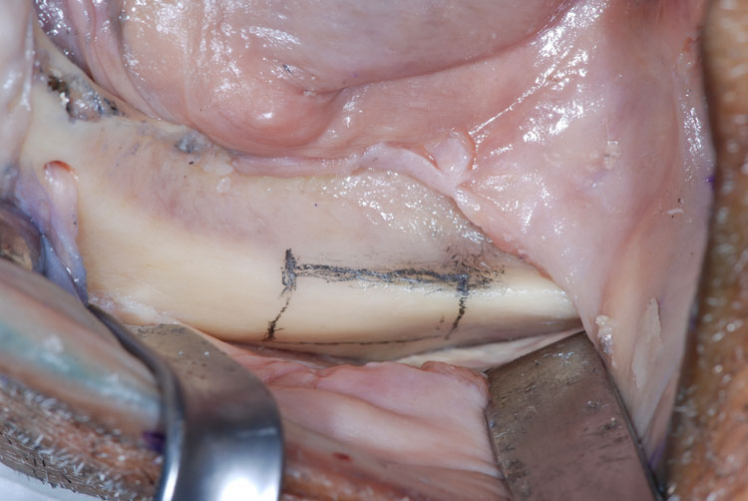
A harvesting area of about 2 cm in length, 1 cm in height and 0.5 mm in depth was marked with a sterile pencil.

**Figure 9:**
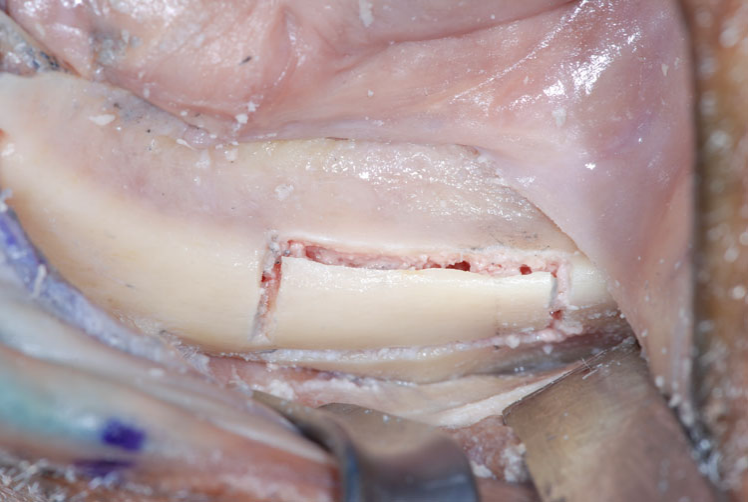
A dedicated piezoelectric insert was used to sculpt the bone shell with copious irrigation with saline solution.

**Figure 10:**
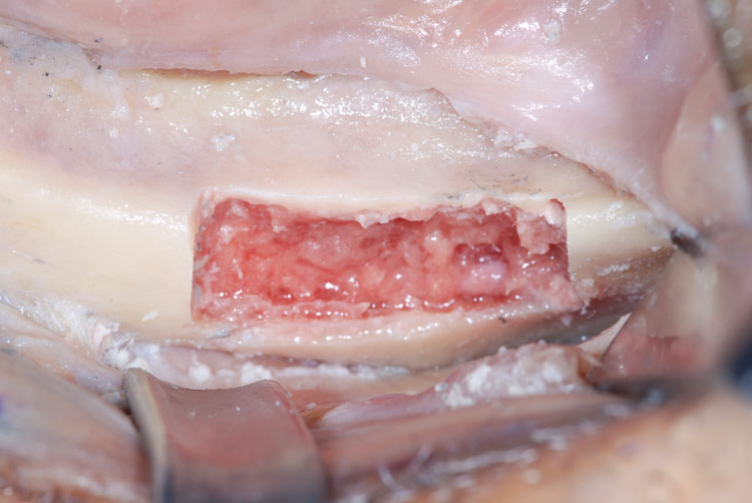
The bone shell was carefully detached from the mandible with a chisel.

**Figure 11:**
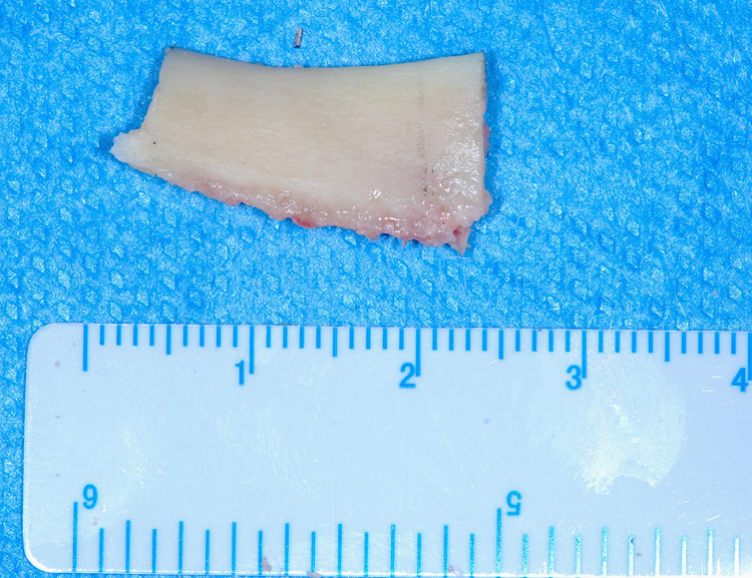
The trapezoidal block had a thickness of about 5 mm and composed of cortical and trabecular bone.

After the reflection of a mucoperiosteal flap, from the first molar to all extension external oblique line of the mandible, an area of 2 cm in length, 1 cm in height was marked with a sterile pencil. A dedicated piezoelectric insert (Ot12, Mectron, Italy) was used to sculpt the bone shell that was detached from the mandible with a chisel. The block had a thickness of about 5 mm and composed of cortical and trabecular bone (Figures 7, 8, 9, 10, 11).

#### 2.1.3 Grafting

**Figure 12:**
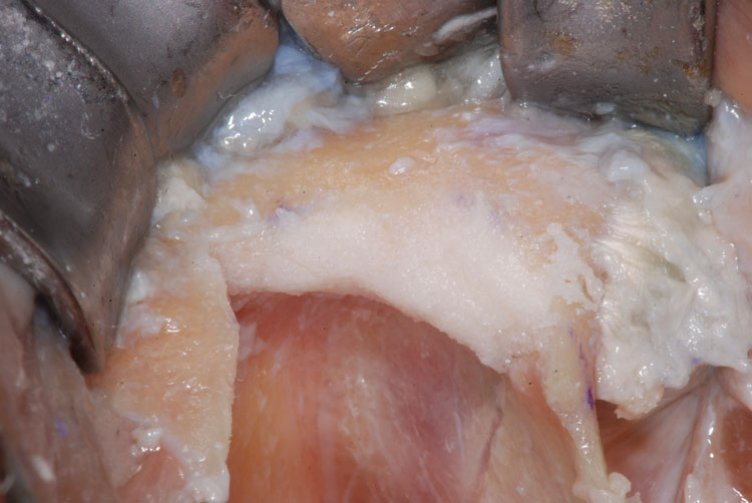
The zygomatic bone defects were filled by autologous bone particles.

**Figure 13:**
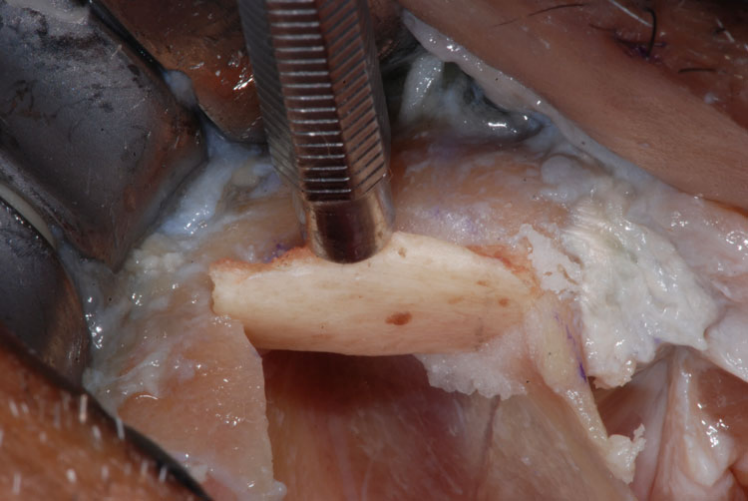
The recipient site was prepared to house the bone shell and allow its primary mechanical retention.

**Figure 14:**
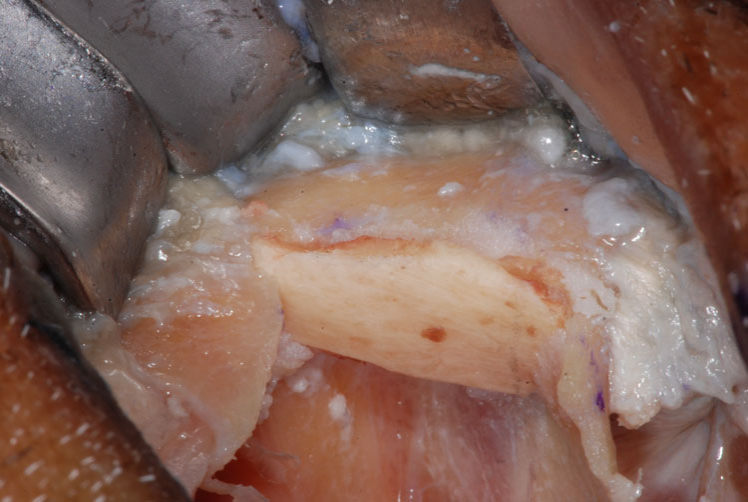
After having carved the recipient site, the adapted the bone shell, appeared stable and with a seamless integration between the mandibular cortical bone and the surrounding zygomatic bone.

**Figure 15:**
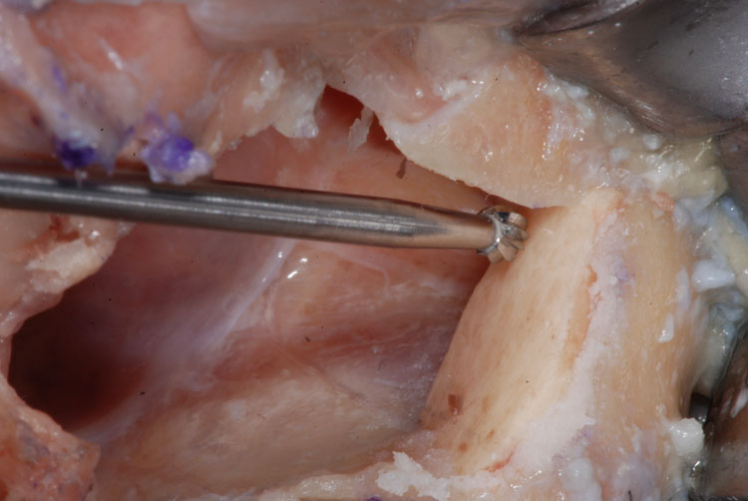
The recipient site of the bone shell fixation screw was prepared by means of a Rounded Drill Ø 1.8 to create a notch on the shell surface.

**Figure 16:**
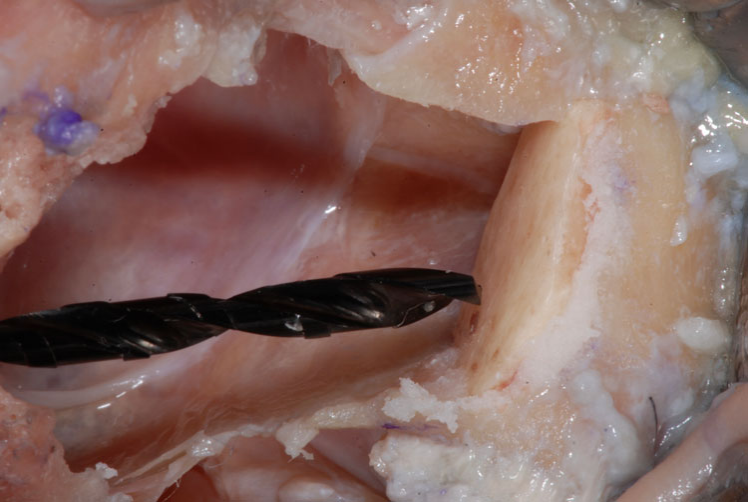
Then a Twist Drill Ø at the Tip of 2 mm was used to countersink and prepare the housing of the screw head.

**Figure 17:**
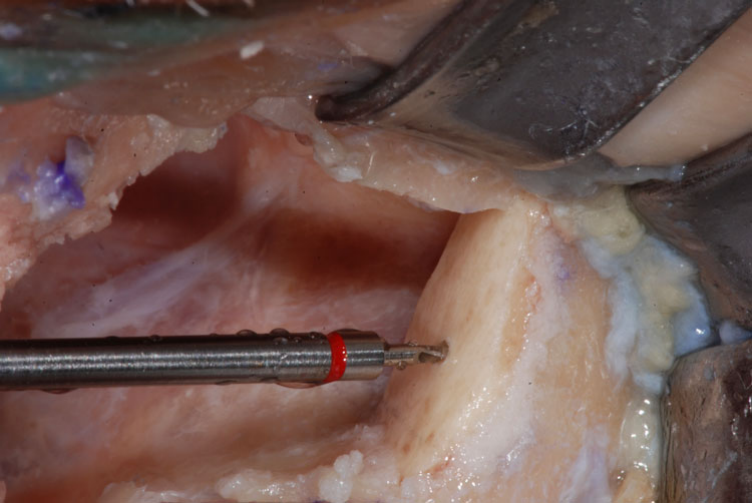
Finally, a Guided Twist Drill Ø 1.2 × 20 mm was used to go through the bone shell up to engaging the zygomatic bone to achieve a bi‐cortical anchorage of the fixation screw.

**Figure 18:**
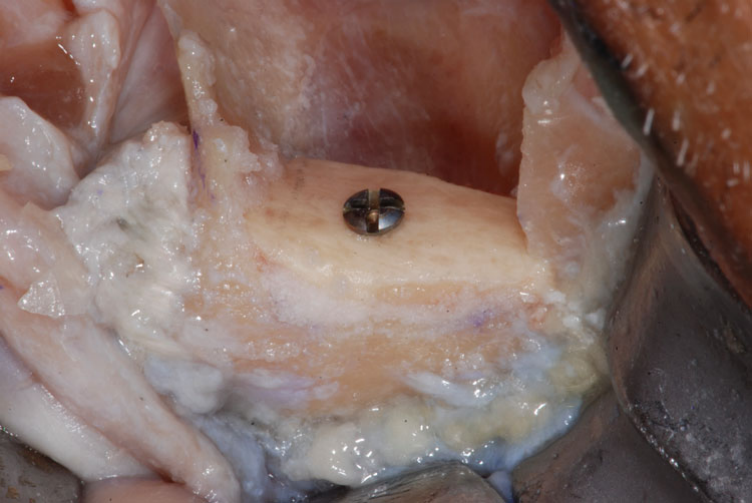
The fixation screw was placed in the center of the graft, to ensure excellent stability in the inter‐defect septum and allow a bi‐cortical contact between the graft and the residual zygomatic bone.

The recipient site was prepared to house the bone shell and allow its primary mechanical retention. The zygomatic bone defects were filled by autologous bone particles before securing the shell with a self‐tapping bone fixation screw (creos bone fixation screws, 1.5 × 12 mm, NobelBiocare, Switzerland). The recipient site of the screw was prepared by means of a Rounded Drill Ø 1.8 to create a notch on the shell surface, a Twist Drill with Tip 2 mm × 7–10 mm to countersink and prepare the housing of the screw head, and Guided Twist Drill Ø 1.2 × 20 mm to achieve a bi‐cortical anchorage of the screw. The fixation screw was placed in the center of the graft, to ensure excellent stability in the inter‐defect septum and allow a bi‐cortical contact between the graft and the residual zygomatic bone (Figures 12, 13, 14, 15, 16, 17, 18). The zygomatic bone graft was protected by the buccal fat pad.

#### 2.1.4 Suturing

The suturing of the donor and recipient sites was achieved with a 3‐0 PTFE suture after detaching the muscular insertion and releasing the tension from the flaps.

## 3 Discussion

The zygomatic bone, comprising 98% of a cortical component and minimal trabecular tissue, presents a viable therapeutic option for implant‐supported rehabilitations. Unlike the maxillary bone, the zygomatic bone is less susceptible to resorption, making it suitable for implant support (Brånemark et al. 2004). A failing zygomatic implant and even more a quad zygoma failure can result in a severe bone defect affecting the entire height of the zygomatic bone pyramid. Such bone deformities may infringe the immediate and delayed placement of new ZIs, requiring complex surgical procedures to restore the integrity of the zygomatic bone anatomy.

This article aims to present a proof‐of‐concept surgical technique and illustrate its clinical application on a cadaver specimen for the immediate reconstruction of zygomatic bone after ZI failure and the related complications. The three‐dimensional reconstruction of zygomatic bone defect was achieved by a specific form of guided bone regeneration or shell technique, using a thin cortical plate harvested from external oblique line of the mandible.

The Zygoma Bone Shell technique should be considered where the failure of zygomatic implants has resulted in a significant destruction of the supporting bone, rendering the placement of a new implant particularly challenging. The bone shell surgical technique was introduced to address minor horizontal and vertical bone defects and its favorable application to zygomatic implant failure is tightly dependent on the extension of the defect. Even though composite bone shells reconstruction with multiple bone laminas may be used in larger defects.

Autogenous bone graft is osteoinductive, osteogenic and osteoconductive, with significant regeneration capacity, representing the gold standard in case of large augmentation procedures (Misch 2000, 1997; Pozzi and Mura 2014). However, the most serious problems with autogenous full block transplants reported are the resorption rates of 21%‐25%. The shell technique according to Khoury was developed to circumvent this problem (Khoury and Hanser 2015). The “three‐dimensional” reconstruction of the shell technique determined an accelerated vascularization in the bone box and the greater volume stability of the avascular cortical thin plate reduces bone resorption to under 10% (Khoury and Hanser 2015).

In cases of zygomatic implant failure, various therapeutic alternatives have been proposed. Hirano et al. (Rigolizzo et al. 2005). demonstrated successful reconstruction using hydroxyapatite, after surgical removal of intraosseous hemangioma in the zygomatic bone. However, this technique may not be applicable in the absence of bone peaks and containing defects resulting from zygomatic implant failure.

Xue et al. (Hirano et al. 1997). utilized 3D printing for orbital‐maxillary‐zygomatic reconstruction, providing precise preoperative planning and accurate postoperative results. While effective, this technique may be more complex than the proposed protocol.

Chu et al. (Xue et al. 2019). evaluated the efficacy of CAD/CAM techniques in reconstructing complex zygomatic defects, achieving consistent results. However, the complexity and applicability of this protocol should be considered, and the need to stage the reconstruction after the implant removal to execute a cone beam computed tomography (CBCT) that allows the ideal 3D visibility of the zygomatic defect.

Extra‐oral grafting approaches as single free fibula flap microsurgery (Chu et al. 2012), and computer‐assisted zygoma reconstruction with vascularized iliac crest bone graft (Heredia‐Alcalde et al. 2021), should be considered only to address extensive defect interesting also the maxillary bone and the zygomatic arch.

Mommaerts et al. (Modabber et al. 2013). discussed challenges in achieving perfect adaptation of the zygomatic grafts, emphasizing the importance of symmetrical shaping and fixation to prevent complications. Intraoral mandibular harvesting emerged as a preferential surgical option to address complications associated with zygomatic implant failure, bringing forth a range of distinctive advantages. A pivotal consideration is the diminished incidence of postoperative complications associated with intraoral mandibular harvesting (Vandeputte et al. 2020). Furthermore, the increased convenience for the patient is a noteworthy consideration. The intraoral nature of mandibular harvesting reduces the invasiveness of the procedure, enhancing patient comfort and expediting recovery times. This characteristic could substantially contribute to rendering the overall surgical experience more manageable for individuals undergoing the treatment.

Finally, the favorable aspect is the comparable composition between mandibular and zygomatic bone, particularly in the cortical region. This anatomical resemblance facilitates optimal structural compatibility, fostering seamless integration of the bone graft into the zygomatic area (Mommaerts et al. 1995).

## 4 Conclusion

Within the limitations of this proof‐of‐concept, the zygoma bone shell technique may offer a viable surgical procedure for immediate bone reconstruction after zygomatic implant failure. Translating the previously reported clinical outcomes of bone shell technique, it may be used same day of failing implant removal to achieve reconstruction of zygomatic anatomy with limited risk of postoperative complications. The achieved three‐dimensional reconstruction of the zygomatic anatomy could offer a precise solution with potential low risk of postoperative complications. Further clinical studies are needed to confirm its predictability, reliability and anticipated patient benefits.

## Author Contributions

E.L.A and A.P. conceived study aims and design, and developed the surgical technique E.G led the writing.

## Conflicts of Interest

The authors declare no conflicts of interest.

## Data Availability

According to the University Institutions regulations, study data are in the University repository and not publicly available to avoid compromising ethical standards and legal requirements. However, study data may be available on request from the corresponding author in respect of privacy and ethical restrictions. The study execution was followed by the Scientific Boards of the Dental Schools of Vita Salute University, San Raffaele Hospital, Milan, Italy and University of Rome Tor Vergata. For this type of study: a proof‐of‐concept surgical technique description for the immediate reconstruction of zygomatic bone following ZI failure and complications, illustrating the related clinical steps in a cadaver specimen was not required by the two universities any Ethical Committee Approval.
